# Impact of D-Amino Acids in Schizophrenia

**DOI:** 10.3390/biom15091270

**Published:** 2025-09-02

**Authors:** Serdar M. Dursun, Leman H. Dursun, Glen B. Baker

**Affiliations:** Department of Psychiatry (Neurochemical Research Unit and Bebensee Schizophrenia Research Unit) and Neuroscience and Mental Health Institute, University of Alberta, Edmonton, AB T6G 2B7, Canada; dursun@ualberta.ca (S.M.D.); leman.dursun23@gmail.com (L.H.D.)

**Keywords:** amino acids, D-serine, D-aspartate, D-alanine, D-cysteine, schizophrenia, serine racemase, D-amino acid oxidase, D-aspartate oxidase

## Abstract

Most amino acids contain a chiral center and thus, can exist as L- and D-isomers. For many years, it was thought that only the L-isomers were present in mammals. However, in recent decades it has been demonstrated that D-isomers are also present. Three of these amino acids, namely D-serine, D-aspartate, and D-alanine, have been proposed to play a role in the etiology of schizophrenia via interactions with glutamate receptors. D-Serine and D-alanine act at the glycine modulatory site on the NMDA receptor, while D-aspartate acts at the glutamate site on the same receptor. D-aspartate also acts on the mGlu5 receptor and can stimulate glutamate release presynaptically. Preclinical studies have reported that manipulations to reduce brain levels of D-serine, D-aspartate, or D-alanine lead to schizophrenia-relevant behaviors, and clinical studies have reported reduced levels of these D-amino acids in the brain tissue (postmortem) and/or body fluids from schizophrenia patients compared to those noted in controls, although there are some contradictory findings. The possible use of these amino acids and/or the manipulation of their relevant enzymes in the treatment of schizophrenia are described. D-Cysteine has been identified recently in human brain tissue, with the highest values in white matter; demonstration of its involvement in brain development has led to speculation that it could be involved in the etiology of schizophrenia, identifying it as a potential therapy in combination with antipsychotics. Future directions and potential problems that should be considered in studies on D-amino acids and their relevant enzymes in schizophrenia are discussed.

## 1. Introduction

Schizophrenia, a devastating neurodevelopmental disorder, exhibits a worldwide prevalence of approximately 0.7–1.0% [1,2]. It is a heterogenous disorder characterized by the presence of symptoms denoted as positive (hallucinations, delusions, disorganized thinking and behavior) and negative (blunted affect, alogia, avolition, difficulty with social interactions), as well as cognitive impairment [3,4,5,6]. Schizophrenia is a complex disorder considered to involve interactions among multiple genetic, epigenetic, and environmental factors [5,6,7,8,9,10,11,12,13]. There are at least three phases of the disorder, including the prodromal phase, the initial onset of psychosis, and the chronic illness [14,15]. Although the dopamine hypothesis [16,17,18,19,20,21,22,23] remains an important theory of schizophrenia that has also been valuable in the development of antipsychotic drugs, many people with schizophrenia do not respond well to the current antipsychotics, and it is evident that other factors are also important in the etiology of this heterogenous disorder, including dysregulation of other neurotransmitters and neuromodulators in addition to dopamine. These other neurotransmitters and neuromodulators include glutamate [24,25,26,27], 5-hydroxytryptamine (5-HT, serotonin) [25,28,29], γ-aminobutyric acid (GABA) [30], acetylcholine [31], and D-serine [32]. There is a large body of evidence indicating that schizophrenia is a very complex disorder and that in addition to neurotransmitter/neuromodulator dysregulation, other contributing factors include genetics/epigenetics [5,6,11,12,13], the immune system [9,33], neuroendocrine function [33,34,35], oxidative stress [36], mitochondrial dysfunction [37], the gut–brain axis [38,39,40,41,42], and the blood–brain barrier [10,43], with glia as well as neurons playing important roles, [44,45].

The focus of the current review article is on the relationship of the free D-amino acids to the etiology, diagnosis, and possible treatment of schizophrenia. Many amino acids contain a chiral center and consequently can exist as L- and D-isomers. For many years, it was considered that only the L-isomers of these amino acids existed in mammals, but in recent decades, it was discovered that measurable amounts of D-isomers of several amino acids were also present, although usually at considerably lower levels than those of the corresponding L-isomers [46]. Three of these, namely D-serine, D-aspartate, and D-alanine, act on N-methyl-D-aspartate receptors (NMDARs) and have been under investigation for their potential role in the etiology of schizophrenia and other neuropsychiatric disorders, including Alzheimer’s disease [46,47,48]. They are of particular interest with regard to schizophrenia since they interact with glutamate receptors, and the glutamate hypothesis of schizophrenia, in which there is hypofunction of NMDARs, is now a widely accepted hypothesis [49,50,51]. D-Cysteine, which has recently been identified in the mammalian brain [52], binds to myristoylated alanine-rich protein kinase C (MARCKS) and has been proposed to be a physiological regulator of the proliferation of neural progenitor cells (NPCs) by inhibiting AKT serine–threonine protein kinase signaling mediated by FOXO1 and FOXO3a, and has been proposed to play a potential role in schizophrenia, a neurodevelopmental disorder [53].

The literature search related to this manuscript was conducted using the PubMed, Web of Science, PsychInfo, and Scopus databases, entering the phrase, “D-amino acids in schizophrenia”, as well as a similar phrase for each of D-serine, D-aspartate, D-alanine, and D-cysteine in schizophrenia, searching from 1980 to the present. Only papers written in English were included. Relevant review articles were also searched for additional papers related to these D-amino acids.

Most of the interest in D-amino acids in schizophrenia has focused on D-serine, a potent co-agonist at the glycine modulatory site on the NMDAR. This interest in D-serine also stimulated subsequent research on other D-amino acids in schizophrenia, notably D-aspartate and D-alanine. Although the D-isomers of several other amino acids may also have a role to play in schizophrenia and/or other disorders [46], free D-serine, D-aspartate, D-alanine, and D-cysteine are the focus of this review paper.

## 2. D-Serine

**Figure 1:**
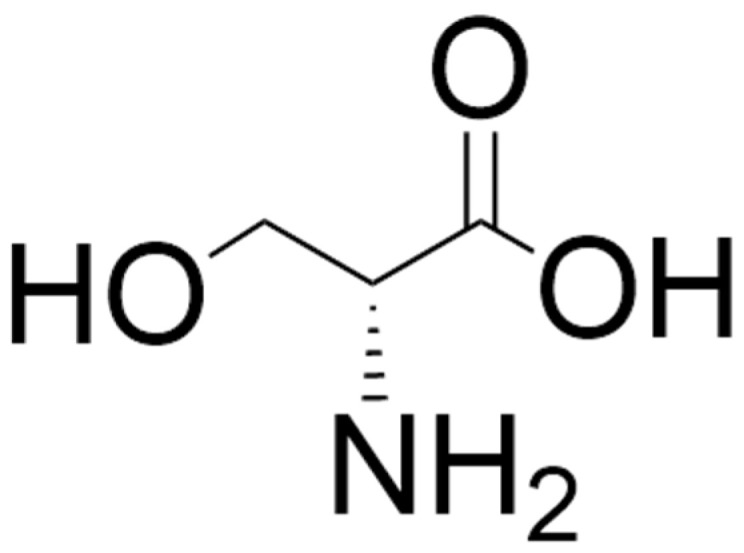
Structure of D-serine. The structure was located by a Google search and drawn using ChemDraw (version 25.0).

**Figure 2:**
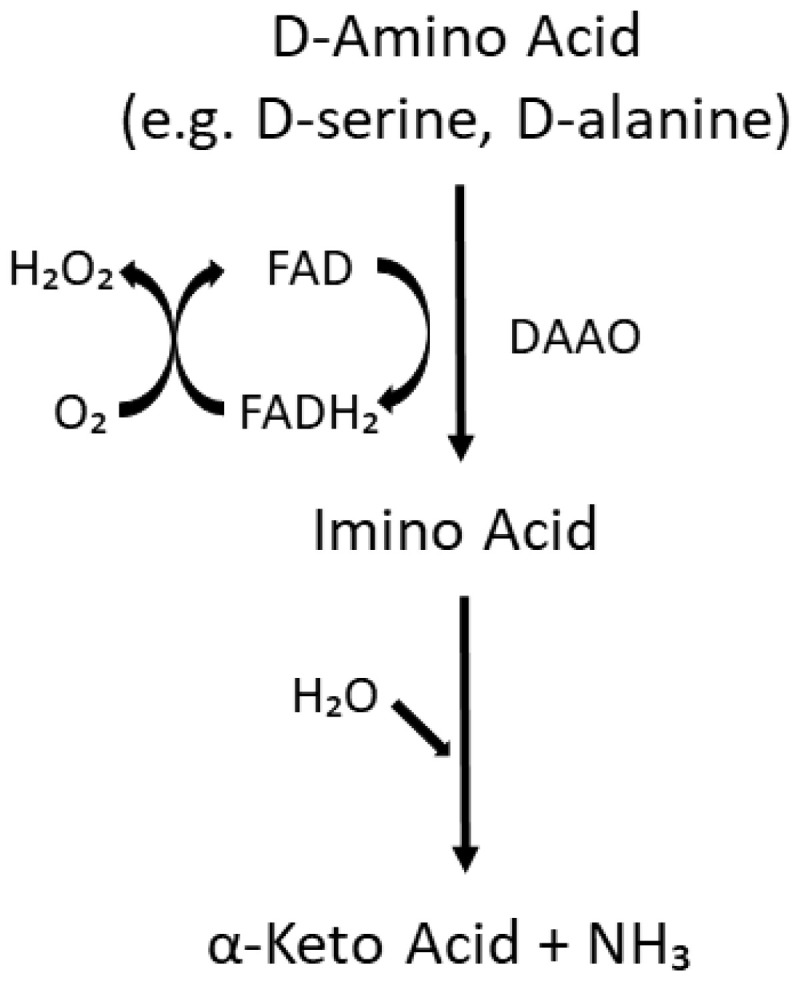
Enzymatic pathway catalyzed by DAAO. Substrates include D-amino acids with small, neutral side chains such as D-serine, D-alanine, D-proline, and D-leucine [77]. FAD = flavin adenine dinucleotide. This figure was drawn using PowerPoint and was adapted based on information available in the literature (e.g. [32,77,78]).

Of the D-amino acids, D-serine (Figure 1) has been the most extensively studied in psychiatry. In the 1990s, it was shown that free D-serine is present in the mammalian brain [54,55,56]. This D-amino acid is enriched in brain areas with high levels of NMDARs such as the cerebral cortex and hippocampus [57] and has been shown to be a potent co-agonist at the glycine modulatory site on NMDARs and to be involved in neurotransmission, neurotoxicity, synaptic plasticity, cell migration, and regulation of sleep [58,59,60,61,62,63,64,65,66,67,68]. The principal enzymes involved in the formation and catabolism of D-serine are serine racemase (SR) and D-amino acid oxidase (DAAO), respectively [32,59,69,70,71,72,73]. SR can also catalyze the degradation of D-serine through an α,β-elimination mechanism, which may be involved in the regulation of intracellular D-serine levels [74]. A schematic of the reaction catalyzed by DAAO is shown in Figure 2.

Diet and the gut microbiota can also contribute to D-serine levels in the body [46,47]. Interestingly, Osaki et al. [75] have reported that the 10% of the D-serine in the brain that is not removed in SR knockout mice is not affected by diet restriction or gut microbes, suggesting the existence of an enzyme in addition to SR involved in the formation of D-serine. The regional distribution of SR in the brain is similar to that of D-serine, i.e., high levels in the cortex and hippocampus and much lower levels in the brain stem and cerebellum [32]. The opposite situation exists for DAAO, with this enzyme present in the brain stem and cerebellum in high concentrations, but at much lower levels in the prefrontal cortex (PFC), hippocampus, and substantia nigra [32,72,76,77,78,79].

Several research groups have reported decreased serum, plasma, or cerebrospinal fluid (CSF) levels of D-serine in schizophrenia patients compared to in healthy controls [80,81,82,83,84,85,86,87,88,89], although there are some contradictory findings [90,91,92,93]. Rampino et al. [94] have pointed out the importance of studying the phases of schizophrenia, since they determined that serum levels of D-serine and the D-serine/total serine ratio were significantly higher in people at high risk for developing psychosis than in healthy controls or in people with full-blown schizophrenia. Patients in the latter category displayed significantly lower levels of the D-serine/total serine ratio than healthy controls, high-risk people, and patients with first-episode schizophrenia. Uysal et al. [88], in a study on first episode schizophrenia patients who had not taken antipsychotics prior to the study, found that prior to treatment the patients had lower levels of D-serine, DAAO, and D-serine/DAAO ratio than healthy controls, but that after six months of treatment, the levels of D-serine and DAAO were higher in these patients, but at levels still lower than those in the controls. These researchers found no correlation between serum levels of D-serine, DAAO, or D-serine/DAAO and cognitive function. There have been several investigations on the use of D-serine, alone or in combination with antipsychotics, for the potential treatment of schizophrenia [95,96,97,98,99,100,101]. Improvement in positive, negative, and cognitive symptoms with D-serine have been reported, but there have also been less promising results described [102,103,104,105,106]. Tsai et al. [107] proposed that the lack of effect of D-serine in clozapine-treated patients may have been due to the agonist or partial agonist effect of clozapine at the NMDARs or the more severe pathology of patients being treated with clozapine. Although clinical trials have reported a good safety profile with D-serine, because of the possibility of peripheral neuropathies or acute tubular necrosis at high doses of D-serine [32,72,108] several researchers have proposed using DAAO inhibitors as potential antipsychotics (see discussion five paragraphs below).

There has been a great deal of research conducted on the large number of factors that regulate SR activity [32,109,110]. Preclinical studies on the depletion or inhibition of SR in rodents have reported lowered brain levels of D-serine and the production of schizophrenia-like behaviors [32,54,55,76,111,112,113,114,115,116,117,118]. In a recent publication, Lahogue et al. [119] conducted a study on a two-hit model in mice combining deletion of SR and maternal separation; these mice displayed increased locomotor activity (mimicking positive symptoms in humans), memory impairment, and cognitive deficits. Arizanovska et al. [120] have provided an extensive discussion of studies of D-serine across species and concluded that D-serine is a potential therapeutic target to improve sociability, cognition (particularly hippocampal learning and memory), and sleep. Hagiwara et al. [118] treated neonatal mice with an inhibitor of SR and observed behaviors in juvenile (5–6 weeks old) and adult (10–12 weeks old) mice; they reported reduced brain levels of D-serine and the appearance of behaviors relevant to prodromal symptoms and adult symptoms of schizophrenia. These researchers found an improvement in prepulse inhibition (PPI) deficits in the adult mice after an early single dose of D-serine.

*DISC1* is a gene that encodes the scaffold protein DISC1, which in turn is involved in multiple processes that are important for brain health but which deteriorate in several neuropsychiatric disorders. These processes include cell proliferation and differentiation, mitochondrial transport, neuronal growth, synaptic pruning, glutamate receptor trafficking, and dopamine signaling [121]. It has been proposed as a risk factor for several psychiatric disorders, including schizophrenia, although its relative importance as a risk factor remains a matter of debate [121]. DISC1 binds to SR, and it has been reported that disruption of the SR–DISC1 complex results in production of schizophrenia-like behavior via depletion of D-serine [122,123].

Although pharmacological inhibitors of SR are available [124,125,126], there is a paucity of SR enhancers [127,128]. In a recent investigation, Lu et al. [129] found that the tannic acid analogue dodecagalloyl (α12G) produced an 8-fold increase in racemization activity of human SR, but at high doses, it inhibited SR. In studies on administration of α12G to the dizocilpine (MK-801)-treated mouse model of schizophrenia, Lu et al. [129] also reported that this drug improved behaviors associated with NMDAR hypofunction and related to positive symptoms and cognitive impairment in schizophrenia.

Results of studies on protein expression of SR in schizophrenia patients have been inconsistent, with decreases, increases, and no change compared to the results for healthy controls reported [32].

Studies in mice lacking DAAO reported improvement in a number of behaviors related to schizophrenia [32,72,130,131], suggesting that DAAO inhibitors might be useful in the treatment of schizophrenia. Maekawa et al. [130], in a study of mutant mice lacking DAAO, found that hippocampal long-term potentiation (LTP) and Morris water maze learning were facilitated in the mutant mice relative to wild-type mice. In a behavioral and biochemical study in ddY/DAO mice, which lack DAAO activity, Almond et al. [131] reported increased occupancy of the NMDAR glycine site and enhanced NMDAR function in vivo in the mutant mice. Labrie et al. [72] compared wild type mice and mice carrying the hypofunctional Dao/^GIBIR^ mutation and found that the mutant mice exhibited enhanced adaptive learning in response to changing conditions. These investigators also measured D-serine levels in brain regions in the two groups of mice and reported that D-serine levels were increased slightly in the whole cortex and hippocampus, markedly in the cerebellum, and not at all in the PFC or amygdala in the mutant mice. Other studies in rodents lacking DAAO have also reported small or no increases in levels of D-serine in the forebrain and very large increases in the hindbrain [55,132,133,134,135]. Rais et al. [136] studied the pharmacokinetics of orally administered D-serine in DAAO knockout and wild type mice and found that plasma D-serine levels were markedly sustained in the mutant compared to the wild type mice and that co-administration of D-serine and a DAAO inhibitor in wild type mice enhanced D-serine levels. They surmised that a long-life DAAO inhibitor should be useful in treatment of schizophrenia since it would be able to maintain high plasma D-serine levels over a sustained period of time. In a study on astrocyte-specific DAAO-conditioned knockout mice, Gonda et al. [135] reported elimination of DAAO expression in the hindbrain and a significant increase of D-serine levels in the cerebellum, but not in the forebrain or periphery. They concluded that astrocytic DAAO is involved in regulation of D-serine levels in the hindbrain and hypothesized that brain pathology has no influence on levels of D-serine in blood or urine. Several studies in animal models of schizophrenia have reported antipsychotic-like characteristics of the DAAO inhibitor sodium benzoate [73,137,138,139], a food preservative with antifungal and antibacterial properties [140,141]. Matsuura et al. [137], in a phencyclidine (PCP) model of schizophrenia in mice, found that pretreatment with sodium benzoate attenuated PPI deficits and hyperlocomotion and did not alter levels of D-serine in the plasma, frontal cortex, hippocampus, or striatum. These researchers did not measure levels of D-serine in the cerebellum. Sershen et al. [73], in a PCP-treated mouse model, reported that pretreatment with sodium benzoate produced a reduction in PCP-induced locomotor activity but did not affect plasma or whole brain levels of D-serine. Mahmoud et al. [138] found positive effects on daily life activities, spatial learning, and working memory in rats receiving ketamine when the rats were pretreated with sodium benzoate. They also reported some worsening of liver function with sodium benzoate in this animal model; blood or brain levels of D-serine were not measured. In a ketamine-treated rat model of schizophrenia, Huang et al. [139] reported that sodium benzoate pretreatment resulted in antipsychotic-like behavioral effects in rats but did not alter levels of D-serine or D-alanine in the PFC or hippocampus.

Consistent reports of marked increases in levels of D-serine in the cerebellum but not in the forebrain raise questions about the role of D-serine in the reduction of schizophrenia-like behavior observed in these rodents lacking DAAO. It is interesting that there is considerable evidence that dysfunction of the cerebellum is involved in schizophrenia psychopathology, particularly in cognitive dysfunction [142]. In addition, as mentioned by de Oliveira Souza et al. [143] when discussing the apparent lack of effect of DAAO inhibitors on D-serine brain levels, DAAO appears to be a metabolic hub for the production of other neuromodulators as well, including hydrogen sulfide (H_2_S) and kynurenic acid.

An association between the *DAAO* gene and the gene for G72, a proposed regulator of DAAO, has been reported in schizophrenia [144,145,146,147,148,149,150,151], although there are some disagreements in the literature about these findings [32,151,152]. *G72* is a primate-specific gene that encodes pLG72 protein, which is a modulator of DAAO, although the nature of that modulation is controversial [32,151,153]. Increases [149,150,154], decreases [155], and no difference [156] in expression or protein levels of G72 in schizophrenia patients compared to results for healthy controls have been reported. Lin et al. [157] used computational artificial intelligence and machine learning tools to differentiate schizophrenia patients from healthy controls using G72 single nucleotide polymorphisms (SNPs) and G72 plasma protein levels and concluded that G72 protein alone, without the two SNPs, may have been sufficient to identify the schizophrenia patients. In a study of 355 schizophrenia patients and 86 healthy controls in the Taiwanese population, Lin et al. [158] found that plasma levels of both DAAO protein and G72 protein were higher in the schizophrenia patients. This finding for DAAO is in contrast to the findings of Uysal et al. [88], who recently reported lower serum DAAO levels in first-episode schizophrenia patients than in controls. In a recent publication, Yilmaz et al. [155] studied the protein levels of G72 in schizophrenia patients (one group of drug-naïve patients and one group of patients in acute psychotic episodes) and healthy controls. They found that plasma levels of G72 were lower in the schizophrenia patients (no difference between the two patient groups) than in the healthy controls and that those levels were inversely correlated with age and symptom severity. However, these researchers also found that Receiver Operating Characteristic (ROC) analysis showed poor distinction between patients and controls and suggested that G72 may not be a reliable marker for schizophrenia [155]. In another recently reported investigation, Lin et al. [159] used an interpretable machine learning (IML) framework employing linear regression, least absolute shrinkage and selection operator (Lasso) models, and generalized additive models (GAMs) in 380 Taiwanese schizophrenia patients. The study incorporated 27 parameters covering demographic variables, clinical assessments and functional and cognitive outcomes, and non-linear relationships between features and studied serum levels of DAAO and plasma protein expression levels of pLG72. Based on linear regression, DAAO levels displayed significant association with scores on the 17-item Hamilton Depression Rating Scale (HAMD17). Using the Lasso model, HAMD17 score, age, working memory, and overall cognitive function (OCF) were associated with DAAO in chronically stable patients, and OCF, the Scale for Assessments of Negative Symptoms 20 Item, Quality of Life Scale (QLS), and category fluency were associated with pLG72 in acutely exacerbated patients. Using GAMs, a nonlinear relationship between category fluency and DAAO in chronically stable patients and between QLS and pLG72 in acutely exacerbated patients was observed.

Increases in expression or activity of DAAO in the cerebellum in comparison to that in the controls have been reported by several groups in studies on postmortem brain tissue from schizophrenia patients [77,160,161]. Increases have also been reported in DAAO mRNA and activity in the hippocampus [162] and cortex [163], respectively, but findings in these regions have been less consistent [32]. Interestingly, expression of DAAO has been reported to be markedly elevated in the CSF-producing choroid plexus epithelial cells in schizophrenia patients [164].

In a study on humans in which the DAAO inhibitor sodium benzoate was used as an adjunctive treatment in schizophrenia patients, Lane et al. [165] found improvement in PANSS total score and neurocognition. However, mixed results were observed in subsequent studies with sodium benzoate. Lin et al. [166] used sodium benzoate in schizophrenia patients stabilized on clozapine but having residual symptoms and reported an improvement in positive and negative symptoms but a lack of effect on cognition. In an add-on treatment to sarcosine in chronic schizophrenia, sodium benzoate was found to improve cognition but not positive or negative symptoms [167]. In another trial, sodium benzoate failed to show efficacy in people with early psychosis, but cognitive changes were not measured [168]. In a systematic review and meta-analysis, Seetharam et al. [169] reported that add-on sodium benzoate improved positive psychotic symptoms but had no effect on negative symptoms, general psychopathology, or total Positive and Negative Syndrome Score (PANSS) results. In another systematic review and meta-analysis, Liang et al. [170] included 10 studies on sodium benzoate in their analysis and reported a positive effect on global cognitive function (with a more pronounced effect in women) and an improvement in positive psychotic symptoms but no effect on negative symptoms. In a study on a case series of 12 schizophrenia patients in which sodium benzoate was added to ongoing antipsychotics for 6 weeks, Victor et al. [171] found an improvement in the PANSS total score and cognitive enhancement. Potential adverse effects of sodium benzoate at high doses have been reviewed by Walczak-Nowicka and Herbet [140] and by Hejazl et al. [141]. The former review [140] also provided a summary of beneficial actions of sodium benzoate. Sodium benzoate is still under clinical development as a potential treatment for schizophrenia by SyneuRx International in Taiwan [31,172].

There has been development and investigation of other potential DAAO inhibitors [71,173,174,175,176]. Luvadaxistat is a selective, potent inhibitor of DAAO [174,177,178] that has been investigated extensively. It has been demonstrated that this drug produces an increase in levels of D-serine in the cerebellum, plasma, and CSF in rodents and in the plasma and CSF of humans [174,178,179], with increased hippocampal LTP noted in mice after sub-chronic administration [179]. Luvadaxistat has been reported to show efficacy in improving cognition and social behavior in several rodent models of schizophrenia [178,180], while in a large phase 2 clinical study (INTERACT Study) in humans, it enhanced cognition, but no effect on positive or negative symptoms was demonstrated [31,181]. However, in September of 2024, Neurocrine Biosciences stated that luvadaxistat failed to meet its primary endpoint as a potential treatment for cognitive impairment in schizophrenia in a second phase 2 study and that the company planned to halt further development of the drug at that time [182]. In a recent meta-analysis of double-blind randomized control trials of DAAO inhibitors added to the regimen of schizophrenia patients on antipsychotics, Chang et al. [183] studied five trials with 530 patients. Four of the trials used sodium benzoate, and one used luvadaxistat. On the basis of studying these five trials, these researchers concluded that DAAO inhibitors improved positive and negative symptoms, general psychopathology, and cognitive function [183]. Subgroup analysis for the sodium benzoate trials showed significant improvement in positive and negative symptoms, while the trial with luvadaxistat did not. The authors suggested that three mechanisms, namely inhibition of DAAO, regulation of immune function, and antioxidant properties, may contribute to the beneficial effects of sodium benzoate [183]. These researchers also proposed that larger studies taking into consideration age, baseline severity, treatment duration, and gender are warranted [183]. Other actions of sodium benzoate which have been suggested to contribute to the favorable effects of sodium benzoate include effects on homocysteine levels, inhibition of activation of NFκB, inhibition of microglial activation, and inhibition of both tryptophan degradation and neopterin production [140].

## 3. D-Aspartate

**Figure 3:**
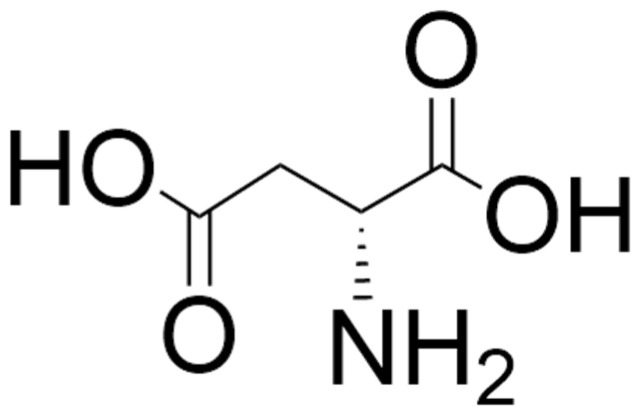
Structure of D-aspartate. The structure was located by a Google search and drawn using ChemDraw (version 25.0).

D-aspartate (Figure 3) activates NMDA receptors by acting as an endogenous agonist at the L-glutamate site of the GluN2 subunit of the NMDA receptor [184,185,186]. This D-amino acid is also thought to exhibit functional involvement with the mGlu5 metabotropic receptor during early neonatal life [184,185] and can evoke presynaptic release of L-glutamate in specific brain areas [186]. D-aspartate was reported to be present in the mammalian brain by Dunlop et al. in 1986 [187]. The synthetic machinery for D-aspartate is still not entirely clear, although an aspartate racemase has been proposed, and SR is also thought to contribute to the formation of D-aspartate from L-aspartate [185,186,188]. However, there is ample evidence that its catabolism is catalyzed by D-aspartate oxidase (DASPO or DDO; EC 1.4.3.3) [189,190]. As with D-serine, some of the D-aspartate in the body comes from the diet and the gut microbiota [47,184,191,192,193,194,195]. While brain D-serine levels elevate postnatally in frontal areas, remain relatively constant in adulthood, and decrease in the elderly (with the possible exception of Alzheimer’s disease patients [196,197,198,199]), D-aspartate levels are relatively high in the developing embryonic brain, with levels decreasing dramatically postnatally with the emergence of DASPO [184,186,200,201]. However, D-aspartate levels in peripheral organs such as the testes, spleen, and pituitary gland increase postnatally and remain relatively constant through adulthood [56,194]. Both D-aspartate and DASPO in the brain are localized predominantly in the neurons, and it has been proposed that in adulthood DASPO is necessary for the removal of D-aspartate where it is no longer required and could, in fact, be neurotoxic if present at high levels for extended periods of time [186,201,202]. Like D-serine, D-aspartate passes the blood–brain barrier efficiently [94,184,186].

In a study on *Ddo* gene knockout mice and in mice treated orally with D-aspartate, Errico et al. [200] found that long-term elevation of D-aspartate levels reduced the deficit in neuronal PPI induced by amphetamine and MK-801, increased NMDAR activity, and caused adaptations of glutamate in the striatum similar to those seen after chronic administration of the antipsychotic haloperidol, producing an enhancement of hippocampal NMDAR-dependent memory. In another study on *Ddo* knockout mice, there was an elevation of D-aspartate levels in the brain, a reduction in motor hyperactivity and of PPI deficits produced by the NMDAR antagonist PCP as well as a decrease in the dysfunctional activation of functional circuits induced by PCP [203]. These same researchers found that *Ddo* mRNA expression is increased in postmortem PFC samples from schizophrenia patients [203]. Studies in D-aspartate-treated rats indicated that increased levels reported of D-aspartate result in enhanced functional connectivity between the hippocampus and the cortex [204]; the same researchers also found that D-aspartate increased spinogenesis in rat hippocampal slices [204]. Sacchi et al. [205], in a study of freely moving mice, that long-term administration of the atypical (second generation) antipsychotic olanzapine, which inhibits DDO, increased extracellular levels of D-aspartate and glutamate in the PFC and that this effect was suppressed in *Ddo* knockout mice. In another set of experiments, these researchers demonstrated, using cortical synaptosomes, that D-aspartate evoked release of L-glutamate through presynaptic stimulation of NMDA, mGlu5, and AMPA/kainate receptors [205]. There are several reports on studies with a knockin mouse model (R26^Dd0/Ddo^) in which *Ddo* is expressed at the zygotic stage, resulting in almost complete elimination of D-aspartate prenatally and postnatally [206,207,208]. Using this model, De Rosa et al. [206] found that despite the dramatic drop in D-aspartate levels (up to 95%), in adulthood, there was not a gross alteration in glutamatergic synapses or myelin basic protein, while the number of GABAergic interneurons in the PFC was increased, and memory performance increased. Grimaldi et al. [207] conducted a comprehensive metabolomic and lipidomic study at several early developmental stages on brains in this knockin mouse model and found changes in levels of several other amino acids (L-threonine, glycine, L-alanine, L-valine, L-glutamate, GABA), of some metabolites involved in brain development and function (choline, creatine, several lipids), of some metabolites relevant to energy metabolism in the brain (glucose, glycerophosphocholine, lactate), and of oxaloacetic acid compared to levels in control mice [207]. Using the heterozygous R26^Ddo/+^ mouse model as a *Ddo* gene duplication model, Lombardo et al. [208] found reduced corticogenesis, reduced cortical and striatal gray matter volume in adults, and the production of social recognition memory deficits at the juvenile phase in these mice. These researchers also conducted a study on a patient with *DDO* gene duplication and found that the D-aspartate/total aspartate ratio (an index of D-aspartate metabolism) was about 2-fold lower in the patient than in normal controls; the patient also displayed severe intellectual disability and thought disorders [208].

Neurochemical studies in postmortem brain tissue from schizophrenia patients have reported increased *DDO* mRNA expression [209], increased DDO activity [210] and reduced D-aspartate levels in the PFC [209,210]. Errico et al. [209], in a study on postmortem brain tissue, reported decreased levels of D-aspartate and its metabolite, NMDA, in the PFC and striatum of schizophrenia patients, and that this decrease correlated with a downregulation of NMDAR subunits in the PFC. Nuzzo et al. [210], in their postmortem study, found that D-aspartate levels were decreased by about 30% in the dorsolateral PFC in schizophrenia patients compared to levels in the controls and that this decrease was associated with a 25% increase in DDO activity. Rampino et al. [94], in their study of at-risk individuals, patients with first episode schizophrenia or full-blown schizophrenia, and healthy controls, suggested that the increased ratio of plasma D-aspartate/total aspartate may be a signature of early stages of progression of psychosis. Garofalo et al. [184] measured serum levels of a number of free amino acids in the serum of patients with treatment-resistant schizophrenia (TRS) and non-treatment-resistant schizophrenia (nTRS), autism spectrum disorder (ASD) patients, and healthy controls. Treatment resistance in the schizophrenia patients was defined as failure to respond to two different antipsychotics, each given for longer than 6 weeks at an optimal dose. These researchers found reductions in serum levels of D-serine and D-aspartate in both groups of schizophrenia patients compared to levels in the controls (but no significant differences between these two patient groups), while there were no significant differences between the ASD patients and controls with regard to amino acid levels [184].

## 4. D-Alanine

**Figure 4:**
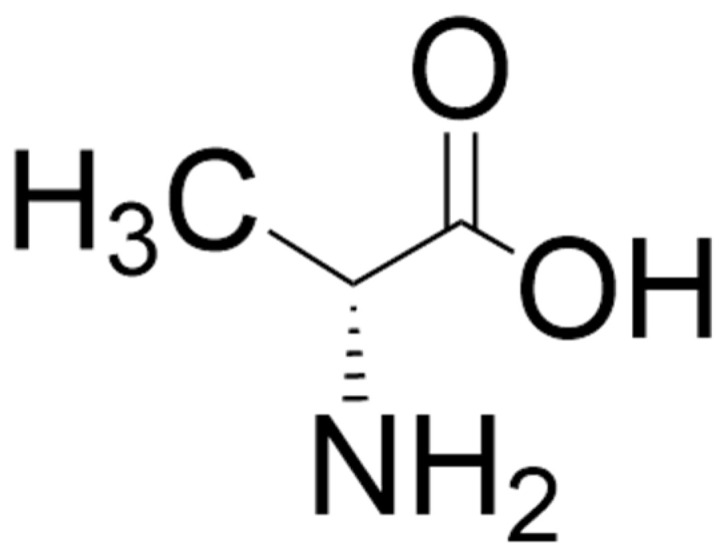
Structure of D-alanine. The structure was located by a Google search and drawn using ChemDraw (version 25.0).

D-alanine (Figure 4) is a potent co-agonist at the glycine binding site of the NMDA receptor [211,212,213,214,215]. The presence of this D-amino acid was first reported in mammals (in the sera of guinea pigs and mice) in the 1960s [216] and was later reported in the brains of mice, rats, and humans (see reference [217] for comprehensive tables of levels of D-alanine in invertebrates, rodents, and humans) and some other higher vertebrates [218]. It is present in relatively high concentrations in the pituitary and pineal glands and in trace amounts in the brain, with particularly low levels in the cerebellum and medulla oblongata [217]. In rats, brain levels of D-alanine have been reported to peak at 6 weeks and then decrease with age [219]. Levels of D-alanine are higher in the pancreas than in the pituitary gland and are reported to be released from the islets of Langerhans by extracellular glucose stimulation [220]. Diet and gut microbiota contribute markedly to levels of D-alanine in mammals [216,217]. Endogenous synthesis, perhaps catalyzed by D-alanine transaminase or alanine racemase, is also likely, but there is still a paucity of information in this regard [217].

It has been reported that levels of D-alanine in mouse pituitary gland, pancreas, and plasma are correlated with circadian rhythm [217,221], with levels of D-alanine during sleeping higher than during waking [217,221,222,223]. D-Alanine is also involved in several other physiological functions and diseases. It has been hypothesized to be a signaling molecule in the gut-brain axis [217]. Blood levels of this D-amino acid have been reported to be decreased in COVID-19 and influenza viral infections [224] and increased in patients with kidney diseases [225]. In rodent models, D-alanine has been reported to be protective against viral infections [217], experimental colitis [226], and acute kidney injury [227]. D-alanine has also been proposed to maintain glucose production via its regulation of circadian rhythm [228].

There is still some uncertainty about the catabolism of D-alanine, with reports that its degradation is catalyzed by DAAO [217] and also reports that it is directly secreted by the kidney, without involvement of DAAO [217]. Several investigations [55,132,133,229,230] have reported that in DAAO knockout rodents, there are increases in levels of D-alanine in several brain regions, peripheral tissues, and body fluids (see reference [217] for a table of results from those studies). Miyoshi et al. reported that in mutant DAAO^-/-^ mice, D-serine values in the hindbrain (cerebellum and medulla oblongata) were much higher than in control mice, but that values in the cerebral cortex, hippocampus, olfactory bulb, hypothalamus, pituitary gland, and liver were not significantly different from control values [230]. In the same study, it was found that D-alanine levels were much higher than control values in the cerebral cortex, hippocampus, olfactory bulb, hypothalamus, cerebellum, medulla oblongata, pituitary gland, and liver [230]. Nagata et al. [229] measured levels of D-serine and D-alanine in the cerebellum and cerebral cortex of mutant mice lacking DAAO and found that D-serine levels were increased markedly in the cerebellum, with no increase over control values seen in the cerebral cortex; in the same mice, levels of D-alanine were much higher in both the cerebellum and cerebral cortex in the mutant mice. In a study on an astrocyte-specific DAAO knockout mouse model, Gonda et al. [135] found that D-serine and D-alanine levels were both significantly higher in the cerebellum of the mutant mice than in controls, while levels of neither of these D-amino acids was increased over control values in the cerebral cortex, kidney, or plasma. Huang et al. [139] administered the DAAO inhibitor sodium benzoate to rats and observed antipsychotic-like effects but no increase in levels of D-alanine or D-serine in the PFC or hippocampus and concluded that the therapeutic effects were independent of levels of either of these two D-amino acids. Rojas et al. [231] reported that administration of D-alanine to monkeys increased plasma and CSF levels of D-alanine, but that administration of experimental DAAO inhibitors had a minimal effect on D-alanine levels. Popiolek et al. [232] reported a lack of effect of administration of the DAAO inhibitor sodium benzoate on D-alanine metabolism in dogs.

Studies on D-alanine administration in rodent models of schizophrenia have reported that it reduces hyperactivity, stereotypy, and ataxia of PCP [233], the accelerating effects on prefrontal metabolism of dopamine [234] induced by PCP and the hyperactivity induced by methamphetamine [235]. Horio et al. [236], in a study in mice, found that coadministration of a DAAO inhibitor with D-alanine (100 mg/kg) reduced PPI deficits induced by the NMDAR antagonist dizocilpine, but that D-alanine, at the same dose alone, had no effect. In the same study, these researchers reported that microdialysis revealed that the combination of the DAAO inhibitor and D-alanine produced a significant increase in extracellular levels of D-alanine in the frontal cortex [236].

Tsai et al. [237] administered D-alanine at 100 mg/kg for 6 weeks to schizophrenia patients as an adjunctive treatment along with various antipsychotics and found an improvement in PANSS-positive, PANSS-cognitive, and Scale for Assessment of Negative Symptoms (SANS) scores, with a favorable side effect profile.

## 5. D-Cysteine

**Figure 5:**
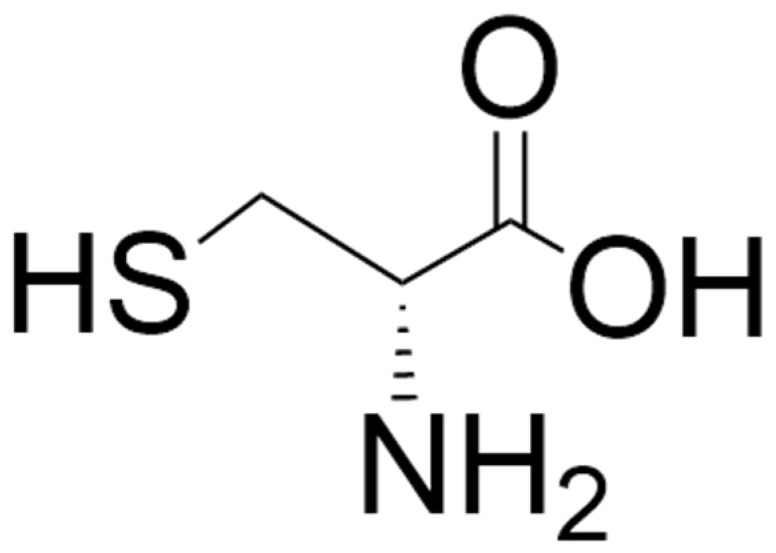
Structure of D-cysteine. The structure was located by a Google search and drawn using ChemDraw (version 25.0).

D-cysteine (Figure 5) is formed from L-cysteine by SR, the enzyme responsible for the formation of D-serine from L-serine [52,53]. It is present in the eye, brain (enriched in PFC, thalamus, striatum, and hippocampus) and pancreas in mice [238,239]. In the human brain, levels of D-cysteine are higher in white matter than in gray matter [239]. D-cysteine has been reported to be present in mM amounts in neonatal brain and decreases to µM amounts progressively with development, suggesting that it has a function in early mammalian neurodevelopment [52,53].

In addition to the endogenous synthesis, it has been proposed that a major source of D-cysteine is the diet [52,53]. In addition to its normal presence in certain foods, food processing procedures can cause non-enzymatic racemization, and L-cysteine undergoes rapid spontaneous racemization [53]. It is thought that regulation of endogenous D-cysteine is performed by the catabolic enzyme DAAO, but that production of H_2_S from D-cysteine may contribute to the regulation of its concentration in some tissues [52,53]; in the latter case, it appears that D-cysteine is converted to mercaptopyruvate (MCP) by DAAO and that conversion of MCP to H_2_S is catalyzed by MCP sulfotransferase [240]. It has been proposed that this H_2_S-forming pathway is localized in the cerebellum and kidneys and is cytoprotective [53]. Through generation of H_2_S, D-cysteine has also been proposed to promote dendritic development [241], to inhibit astrogliosis and microgliosis, and to activate chaperone-mediated autophagy in cerebellar Purkinje cells [242].

A number of properties of D-cysteine indicate that it may be involved in the etiology of schizophrenia [52,53,108]. SR is the biosynthetic enzyme for both D-serine and D-cysteine, and polymorphisms in the SR gene have been reported in schizophrenia patients [83]. Rodents in which SR is depleted demonstrate several schizophrenia-related molecular and behavioral changes [111,113,116,118,119]. MARCKS, a protein involved in neurodevelopment and implicated in schizophrenia [243,244], is a D-cysteine binding protein. Decreased protein expression of MARCKS in the dorsolateral PFC of postmortem brains from elderly schizophrenia patients [243] and an increase in *MARCKS* mRNA in both schizophrenia and bipolar disorder patients [244] have been reported, with a suggestion that this increase could be a compensation for decreased expression of MARCKS protein [53]. MARCKS, which acts as a hub for several signaling pathways, is necessary for normal mouse brain development and postnatal survival [53]. There is an enrichment of MARCKS in neural progenitor cells (NPCs) in the E14.5 mouse brain, and as observed with D-cysteine, there is a decrease in mRNA expression of MARCKS over the course of embryonic and postnatal development [53].

MARCKS is relevant for neuronal survival and migration due to its regulation of the AKT pathway [108]. Findings from numerous preclinical and clinical studies suggest that dysfunction of the AKT signaling pathway is associated with schizophrenia [245,246,247,248,249,250,251,252]. Semenza et al. [52] reported deceased phosphorylation of both AKT1 and MARCKS in growth-factor-starved NPCs. It has also been proposed that FOXO1 and FOXO3a are two downstream targets affected by D-cysteine that can affect cell survival, synaptic plasticity, memory, neural migration, and axon growth [53].

Roychaudhuri and Snyder have raised the question about whether endogenous D-cysteine could be a prognostic biomarker in schizophrenia [53], and de Bartolomeis et al. have suggested that D-cysteine might be a useful agent to add to antipsychotics in the treatment of schizophrenia [108].

## 6. Discussion and Future Directions

Although numerous preclinical studies and clinical trials have now been conducted with D-serine, it is important to obtain more information about the three other D-amino acids described in this review paper. As pointed out in the literature described in this review, there are some contradictory findings with regard to levels of D-amino acids in tissues and body fluids in schizophrenia, in levels and expression of some of the regulators (e.g., AKT) with which they interact, and in possible mechanisms of action of potential new drugs (e.g., sodium benzoate) proposed to act through these amino acids. Numerous important factors should be taken into consideration in future studies on D-amino acids. There are considerable contributions of the diet (natural presence in food and dietary supplements and formation during food processing) and/or the gut microbiota to their levels [46,47,52,53,94,141,184,191,192,193,194,195,217,253,254,255]. Clinical studies on body fluids or postmortem brain tissue should, if possible, include the measurement of levels of all four D-amino acids simultaneously, and age, baseline severity, duration of the illness and of treatment, and gender should be taken into account [81,87,93,170,183]. The potential effects of the antipsychotics and other drugs the patients are taking must be considered [71,85,108,184,202,255,256,257,258,259]. Anti-inflammatory effects, particularly with regard to intestinal inflammation, have been observed with D-serine and D-alanine [47,94,139,226,260,261], but both of these D-amino acids can also elicit proinflammatory responses [255], and further studies on the role of the immune system in the effectiveness of D-amino acids in the treatment of schizophrenia are warranted. Since there are typically several phases of schizophrenia, clinical studies should be conducted over time, i.e., during the prodromal, first episode, and chronic schizophrenia phases [94,184].

There is concern expressed in the literature about understanding more clearly the mechanisms involved in the behaviors produced by the inhibition of DAAO [73,133,134,135,137,139,143,231,232]. In mutant mice lacking DAAO, there are increased levels of D-serine in the hindbrain, kidneys, blood, and urine, but not in the forebrain [131,132,133,134]. A lack of effect on the plasma levels of D-alanine and D-serine in dogs and monkeys by inhibition of DAAO has been reported [231,232,262]. Given the reported discrepancies between inhibition of DAAO and levels of D-serine and D-alanine, it has been proposed that some other factors involved with the enzyme may be relevant [135,139,143]. For example, DAAO also catalyzes the conversion of kynurenine to kynurenic acid [143,161,255,258] and is involved in the production of H_2_S via the catabolism of D-cysteine [143,239,240]. It has also been proposed that other mechanisms such as decreases in plasma levels of L-serine [73] and levels and transport of glycine [73], increases in plasma levels of some metabolites of low molecular weight amino acids [73], antioxidant effects [73,140,170], regulation of immune function [139,170,230,231,232], increased expression of neurotrophic factors, restoration of blood–brain barrier integrity, and effects on sex hormones such as estradiol and follicle-stimulating hormone [259] may be contributing factors in the actions of the DAAO inhibitor sodium benzoate. Studies on this drug in dementia patients have also produced some findings that may have relevance to future studies on schizophrenia patients, particularly since oxidative stress and NMDAR-related dysfunction have been proposed to be involved in the pathogenesis of both disorders [263]. In a study on the effects of 6 weeks of treatment with sodium benzoate or placebo on cognitive function in 97 participants (62 women and 35 men) with behavioral and psychological symptoms of dementia, Lin et al. [264] concluded that treatment with sodium benzoate may improve cognitive function in women with later-phase dementia; it was also reported that when compared with placebo treatment, in these women there was an increase in estradiol to follicle stimulating hormone ratios between baseline and endpoint. In another investigation [263], this time on the effects of sodium benzoate or placebo in patients with mild Alzheimer’s disease, treatment with sodium benzoate at 1000 mg/day resulted in increased activity of the endogenous antioxidant catalase in the plasma in female patients, but not in males. These increases correlated with the improvements in cognition in the women. The researchers concluded that the findings supported the importance of oxidative stress and sex differences in Alzheimer’s disease. In a recent secondary analysis of a randomized clinical trial, Lin and Lane [265] concluded that treatment with sodium benzoate resulted in decreased plasma levels of amyloid beta peptides and improved cognitive function in Alzheimer’s disease patients.

Translation of results from animal models to humans is always a problem when studying schizophrenia, and this problem will also apply to D-amino acids. In addition, brain levels, metabolism, and regional distributions of the D-amino acids may differ somewhat between the animal models themselves and between those models and humans [46,47,73,175,202,266,267], although Arizanovska et al. [120], in a comprehensive review of translationally relevant behaviors across species, have described some helpful similarities between fruit flies, rodents, and humans with regard to the beneficial effects of D-serine.

Although studies on the levels of the D-amino acids, their associated enzymes, and other factors related to their function are relatively easy to conduct in plasma or serum, there is concern about how these levels relate to the levels and physiological effects of D-amino acids in the brain [134,135,143,184]. The D-amino acids have been reported to be present in several peripheral organs and endocrine glands [47,48,52,53,94,112,184,194,217,220,253,254,255,267,268,269,270,271], and those organs and glands may make substantial contributions to the levels of the amino acids. The coexistence of other disorders (comorbidity) with schizophrenia occurs frequently, and these other disorders may confound studies on the D-amino acids in schizophrenia, particularly since D-amino acids have also been proposed to be involved in several such disorders [143,184,202,217,253,254,255,263,266,267,269,270,271].

## 7. Conclusions

Since the discovery in mammals of the four D-amino acids mentioned in this review, there has been a great deal of interest in their potential role in a number of neuropsychiatric disorders. Studies on D-serine, D-aspartate, and D-alanine have increased our knowledge of the functioning of the NMDAR. Investigations in animal models using manipulations of the levels of the D-amino acids and/or of the enzymes involved in their synthesis or degradation have permitted researchers to better understand their possible role in brain function and in the development of schizophrenia-like symptoms, allowing them to design clinical studies investigating their possible roles in the etiology, diagnosis, and therapy of schizophrenia.

Despite various incidences of contradictory findings in the literature in this area and the difficulties mentioned in the Section 6, studies on the potential roles of D-amino acids in normal brain function and in schizophrenia have been exciting, providing clues to the development of possible new therapeutic approaches and/or biomarkers for confronting the challenges of diagnosing and treating this devastating disorder.

## Data Availability

Not Applicable.

## Abbreviations

α-12G	Dodecagalloyl
AKT	AKT serine–threonine protein kinase
ASD	Autism spectrum disorder
CSF	Cerebrospinal fluid
DAAO	D-amino acid oxidase
DASPO;DDO	D-aspartate oxidase
DISC1	Disrupted-in-schizophrenia 1
GABA	γ-aminobutyric acid
GAMs	Generalized additive models
H_2_S	Hydrogen sulfide
HAMD17	17-Item Hamilton Depression Rating Scale
IML	Interpretable machine learning
5-HT	5-Hydroxytryptamine, serotonin
Lasso	Least absolute shrinkage and selection operator
LTP	Long-term potentiation
MARCKS	Myristoylated alanine rich protein kinase C
MCP	Mercaptopyruvate
MK-801	Dizocilpine
NMDA	N-Methyl-D-aspartate
NMDAR	N-Methyl-D-aspartate glutamate receptor
NPCs	Neural progenitor cells
OCF	Overall cognitive function
PANSS	Positive and Negative Syndrome Scale
PCP	Phencyclidine
PFC	Prefrontal cortex
PPI	Prepulse inhibition
QLS	Quality of Life Scale
ROC	Receiver Operating Characteristic
SANS	Scale for Assessment of Negative Symptoms
SR	Serine racemase
SNPs	Single nucleotide polymorphisms
TRS	Treatment-resistant schizophrenia
